# Tandem repeat expansions: the good, the bad and the hidden

**DOI:** 10.1515/medgen-2021-2097

**Published:** 2022-01-12

**Authors:** Christel Depienne

**Affiliations:** Institute of Human Genetics, University Hospital Essen, University of Duisburg-Essen, Essen, Germany


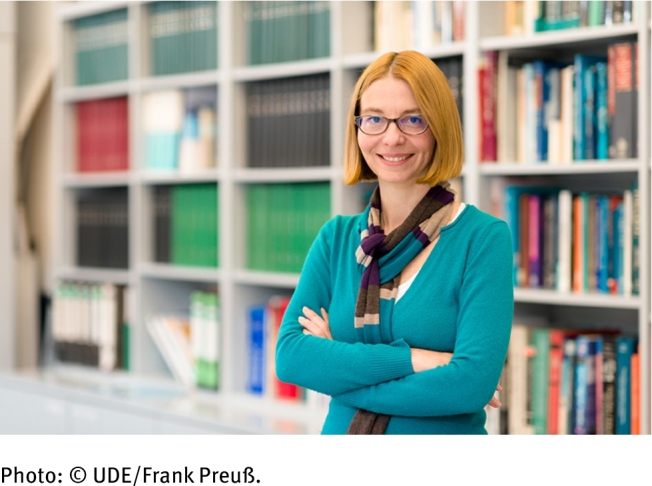


## Fantastic tandem repeats and where to find them

Human genomes contain over a million tandem repeats [1], [2]. These highly instable and polymorphic variations have historically been divided into three groups depending on the size of the repeated motif. Microsatellites (or short tandem repeats, STR) correspond to repetitions of typically 2–6 and up to 9 nucleotides and are the most common repeats present in noncoding regions of genes. Minisatellites (10–99 bp repeats) is a heterogeneous category of repeated elements that is also present but more rarely in specific parts of genes while satellites (≥ 100 bp repeats) are restricted to gene-depleted regions and mainly constitute heterochromatin and centromeres. The distinction between microsatellites and minisatellites is blur and altogether, they form the variable number of tandem repeats (VNTR). Tandem repeats are mainly scattered in noncoding regions of the genome although repetitions of triplets can also be found in a subset of protein coding genes. This rapidly evolving variations are recent in terms of evolution and many of them are even specific to humans. Recent evidence suggests that tandem repeats overlapping regulatory and/or intronic regions could represent functional DNA elements able to regulate or modulate gene transcription [3].

## From normal to pathological

The expansion of tandem repeats across generations is a well-known process described first in 1991 [4], [5], [6] and resulting in at least 50 human disorders [7]. The peripheral and central nervous system and the muscles are the main affected tissues in the vast majority of the expansion disorders known so far, but the examples of Fragile X-associated Primary Ovary Insufficiency (premutation: 55-200 CGG repeat expansions in *FMR1* in females) and Fuchs’s corneal dystrophy (intronic CTG repeats in *TCF4*) suggest that repeat expansions could affect other tissues as well. Because of their repetitive nature, repeats are unstable in a length-dependent manner, with the longest, uninterrupted tandem repeats being by far the most prone to expand. Repeats can expand both during mitosis and meiosis, and their stability/instability is greatly influenced by DNA mismatch repair processes [8].

## Coding or noncoding

Two main types of STR expansions exist: expansions affecting coding regions, mainly associated with codons encoding polyglutamine and polyalanine, and expansions altering non-coding regions of genes. The most frequent coding expansion disorders are associated with CAG triplet expansions, including those found in Huntington’s disease (HD), spinocerebellar ataxias type 1, 2, 3, 6, 7, 17, and are characterized by neuronal intranuclear protein inclusions that contain the polyQ-expanded protein [9]. Noncoding repeat expansions can be located in 5’ or 3’ untranslated (UTR) regions, promoter or introns of genes and they act via distinct mechanisms depending on their location, GC content and impact on gene transcription and translation [7], [10].

## Hunting hidden repeats and discriminating the bad from the benign

The discovery of most repeat expansion disorders has been possible thanks to familial or multiple cases allowing to conduct linkage analyses and has usually taken many years and efforts. Indeed, repeats are difficult to study by short-read sequencing technologies that are still prevalent for clinical applications and repeat expansions are typically missed unless they are specifically looked for by appropriate techniques or bioinformatics tools. The last three years have witnessed an acceleration of repeat expansion discoveries related to the development of both computational tools and third generation sequencing technologies, including long-read sequencing. Yet, despite the technological advances, the study of repeats remains difficult because of their abundance of the human genome, the limited knowledge about their variability in control populations, and the difficulty in distinguishing benign from pathogenic expanded motifs.

In this context, this issue of *Medizinische Genetik* aims to provide an update in the field, featuring selected disorders caused by repeat expansions. The first review by **Arning and Nguyen** provides an update on Huntington disease. It particularly emphasizes the impact of CAG trinucleotide repeat instability, its relationship with DNA repair pathways and how this interaction acts as a modifier of disorder onset and symptom progression. The second review by **Thieme et al.** presents the molecular and clinical characteristics of Cerebellar Ataxia, Neuropathy and Vestibular Areflexia Syndrome (CANVAS), a recessive neurodegenerative disorder clinically recognized in 2011 but which cause, a biallelic AAGGG expansion in *RFC1*, has been identified in 2019. The third review by **Peters et al.** features Familial Adult Myoclonic Epilepsy (FAME) a dominant disorder caused by the same TTTCA repeat insertion in different genes and highlights how repeat expansion can be pathogenic independently of the gene where they occur. The fourth review by **Pozojevic et al.** describes the many facets of X-linked dystonia-parkinsonism (XDP), another neurodegenerative disorder resulting from the insertion of a short interspersed nuclear element (SINE)-VNTR-Alu (SVA) retrotransposon, associated with a polymorphic hexanucleotide (CCCTCT) repeat in *TAF1*. Finally, the last and fifth review by **Schröder et al.** provides an update on GC-rich expansions, which correspond to at least a third of all repeat expansions described so far and highlight the different mechanisms by which these expansions lead to disorder.

We hope that you will enjoy reading these reviews. Beyond their educational purpose, we hope that this issue will raise awareness of the difficulty to identify new pathogenic repeat expansions and the need to use specific techniques or bioinformatic tools in order to look specifically for them. Thirty years after their first description, we have acquired an important knowledge about the molecular mechanisms associated with repeats that can be used, in combination with recent long-read technologies, to systematically study repeat expansions and find those accounting for unsolved genetic disorders, just as any other variant type.
